# If seizures left speechless: CA-P-S C-A-R-E, a proposal of a new ictal language evaluation protocol

**DOI:** 10.1007/s10072-020-04872-x

**Published:** 2020-11-27

**Authors:** Lorenzo Ferri, Luca Vignatelli, Lara Alvisi, Martina Fabbri, Silvia Boscarato, Corrado Zenesini, Laura Licchetta, Lorenzo Muccioli, Paolo Tinuper, Francesca Bisulli

**Affiliations:** 1 Department of Biomedical and NeuroMotor Sciences (DiBiNeM), University of Bologna, Bologna, Italy; 2 IRCCS Istituto delle Scienze Neurologiche Bologna, Bologna, Italy; 3 Alma Mater Studiorum, University of Bologna, Bologna, Italy; 4 Sant’Orsola-Malpighi Hospital, Bologna, Italy

**Keywords:** Epilepsy, Ictal aphasia, Ictal testing, Standardized language protocol

## Abstract

**Introduction:**

We aimed to create standardized protocol for language examination in patients who underwent video-EEG recording and assessed its efficacy in the characterization of ictal language impairment, its ability to differentiate this from impaired awareness, and interobserver reliability in clinical practice.

**Methods:**

From our database of video-EEG recordings, we selected a representative sample of 63 focal seizures with presumed language impairment. A multidisciplinary team of epileptologists, EEG technicians, and speech therapists analyzed the selected videos to highlight the critical issues of ordinary ictal language evaluation. We subsequently followed a multi-step process to develop the protocol and assess its interobserver reliability.

**Results:**

A protocol based on seven tests in hierarchical succession was created, summed up in the acronym CA-P-S C-A-R-E (Closed Answers, Pro-speak question, Simple orders, Common object denomination, Audio repetition, Reading, Evoke). Following its preliminary administration for 5 months, we assessed the inter-observer reliability of 16 healthcare professionals in distinguishing between language impairment and impaired awareness among a sample of 10 seizures, finding a substantial agreement (kappa 0.61).

**Conclusion:**

The proposed protocol, made of simple and easy to memorize tests, is an effective tool that evaluates multiple domains beyond language. Its use could help to recognize ictal aphasia effectively and differentiate it from impaired awareness, minimizing inter-examiner variability.

**Supplementary Information:**

The online version contains supplementary material available at 10.1007/s10072-020-04872-x.

## Introduction

Aphasia is a disturbance produced by the alteration of cortical areas involved in language skill elaboration, usually secondary to vascular, tumoral, or inflammatory lesions that disrupt a cortical network encompassing dominant fronto-temporal-parietal regions and non-dominant temporal-parietal areas [1, 2]. An epileptic discharge involving primary language areas may induce transient and reversible aphasia, which may show similar aphasic manifestations as other aetiologies [3, 4]. The characterization of language deficits during ictal testing should be performed soon after ictal onset, in order to differentiate aphasia from other language/cognitive disturbances, obtaining key localizing and lateralizing information [5]. Indeed, some disturbances such as impaired awareness may be related to seizure propagation and should not be used as localizing signs. These may have a significant impact on diagnostic management and treatment options, especially epilepsy surgery [3], affecting patient outcomes. The characteristics of ictal aphasia are not well elucidated, likely due to the intrinsic difficulty of assessing the various language aspects in the limited time frame related to seizure duration, as well as the use of differing methodologies in many previous reports. To date, only a few studies reported a specific and well-described neuropsychological evaluation of language during epileptic seizures but were limited to non-epileptic convulsive status or were focused on post-ictal language evaluation [6–8]. The latter is the Cincinnati method which consists of presenting, during video-EEG monitoring, a simple sentence through visual channel as soon as a seizure is detected, asking the patient to read the sentence continuously until it is read correctly, in order to detect post-ictal paraphasic errors [6–8]. Nevertheless, to date, there is no standardized protocol specific for ictal language testing. Recently, an ILAE task force developed an ictal testing battery that could allow standardization among different centers [9]. However, the high number of items used to test multiple ictal symptoms and the absence of open-ended questions may limit the efficacy of this battery, especially with very brief seizures and when dysphasia is present [9]. Furthermore, the inter-observer reliability of ILAE protocol has not been systematically evaluated. Based on these premises, we aimed to create a standardized protocol for language examination in patients with suspected ictal aphasia during video-EEG (VEEG) recording and assessed its efficacy in clinical practice.

## Methods

### Protocol creation

#### Patients’ inclusion criteria

We reviewed all ictal VEEG recordings of out- and in-patients referred to our Institute from 1997 to April 2015. An expert EEG technician selected focal seizures in which an apparent language disturbance was present. Seizures with a clear impaired awareness at onset, or that were not tested, and/or in which aphasia was present only post-ictally, were excluded. The VEEG recordings with poor video/audio quality were also excluded. A multidisciplinary team composed of two epileptologists (FB, LF), two speech therapists (MF, SB) and one EEG technician (LA), reviewed the selected VEEG recordings, aiming to discriminate between language disturbances and subtle impaired awareness. In case of disagreement, the VEEGs were excluded from the analysis.

#### Language evaluation

Two speech therapists with expertise in post-stroke aphasia evaluation and rehabilitation, blinded to patient clinical history and EEG features, reviewed the VEEG recordings and, for each patient, selected the most informative ictal VEEG with regard to language disturbance and its testing. The following features were evaluated for each seizure: the pre-ictal patient state (awake, asleep), the mode by which the patient warns EEG technician during the VEEG about seizure onset (verbally, by gestures, pushing an alarm button, or he could not warn but an EEG change could be noted by EEG technician), head orientation during the episode, ictal language characteristics, and post-ictal behavior. Subsequently, the speech therapists transcribed the communicative interaction between patient and examiner (Table 1), allowing a functional evaluation of the effectiveness of words, gestures and verbal pauses in the communicative context [10]. The patients were then divided into three groups: group A if the logopedic analysis revealed comprehension or/and production deficit, group B if a language deficit was identified but could not be better characterized, and group C if it was not possible to reach an ultimate conclusion.

**Table 1:** Example of communicative interaction of patient 9. Eight tasks were administered, of which two through the verbal channel, four with gestural support and two on imitation. The patient complies in all cases, with appearance of echolalia at P4. There is no verbal or mimic-gestural reaction to C.Q.1. A mixed jargon can be appreciated after P2, while in P3, we find echolalia and a neologism. After P7, the answer is consistent and relevant. The oral comprehension does not appear impaired, even if it is not possible to accurately evaluate it because the communication, in many cases, was made up of simple orders with gestural or imitation support. Oral production is affected by a phonological lexicon disorder

Linguistic tests	Operator	Patient
C.Q. 1	how are you?	╧ ø
N.1	what is this, E.? (shows a pencil)	P1 |a pencil↓
C.Q. 2	how do you feel?	P2 |I feel [ad a ‘proke % ‘la: ‘tire % ‘seta ‘proke % ko’sƐ la ‘tua se:¦% sen’tentsa]↑%
C.Q. 3	can you hear me?	P3 |can you hear me [a’dorekmi]↑%
S.O. 1	raise your arms this way (the operator raises her arms)	P4 |to raise your arms this way complies and keeps her arms up
N.2	what is this, E.? (shows a clock)	P5 |Ɛ: % un [‘tjore]↑% Keeps her arms up
S.O. 2	put your arms down	|complies
N.3	what are these? (shows some keys)	P6 |[ad’me: % ad’medi]↑%
C.Q. 4	can you see them? (keeps the keys in front of the patient)	P7 |yes↓
S.O. 3	take them in your hand	| complies
S.O. 4	pass them on the other hand points at the left hand of the patient	| complies
S.O. 5	give them to me (reaches out to the patient)	| complies
S.O. 6	try to flex your legs he/she puts his/her hand close to the patient’s legs	| raises her legs
S.O. 7	flex them to the knee he/she imitates the movement with her own leg and accompanies the patient’s leg	| complies
S.O. 8	the other one, again accompanies the patient’s movement	| complies

#### Protocol development

The multidisciplinary team reviewed the collected data and developed a protocol for ictal language evaluation adapted to the VEEG monitoring setting; attention was given to the examination of critical modalities to be tested and the ability to perform the tests in the limited time frame of the ictal period. The selection of the tasks to be administered was based on current logopedic knowledge; in particular, we used the Italian tests available for the evaluation of stroke patients (Italian-Aachner Aphasie Bedside Test i-AABT and Esame del Linguaggio al Letto del Malato ELLM) [10, 11]. In order to facilitate protocol learning and administration, an ad hoc acronym was conceived, inspired by well-known abbreviations (i.e., ABCD2 score, ABCDE, CHAD-VASc), routinely used in the emergency setting.

### Protocol evaluation and interobserver reliability

The protocol was used from February 2016 to July 2016 to test consecutive seizures by four different EEG technicians during prolonged VEEG monitoring. The EEG technicians were trained to administer the protocol during 2 weeks using simulated seizures developed by multidisciplinary team members. The multidisciplinary team reviewed the recorded seizures in order to assess the efficacy of the protocol in recognizing ictal aphasia and characterizing the observed language deficit.

Subsequently, interobserver reliability of the protocol in distinguishing between language deficit and impaired awareness was evaluated. An afternoon meeting between health professionals having different skills in VEEG monitoring (technicians, residents, epileptologists) was organized at our Department. Each VEEG recording, tested with the proposed protocol, was projected twice in a dedicated room; participants were asked to assess the presence of ictal aphasia and/or impaired awareness in each of the tested seizures. The overall proportion of agreement and interobserver reliability were evaluated for the presence/absence of ictal language deficit and impaired awareness for each pair of observers. Interobserver reliability was calculated by kappa statistics, the ratio of the observed agreement beyond chance to the potential agreement beyond chance, according to the formula of kappa for dichotomous data, and more than two raters, proposed by Fleiss [12, 13]. Kappa value was interpreted according to conventional groups (0.0–0.20 = slight agreement; 0.21–0.40 = fair; 0.41–0.60 = moderate; 0.61–0.80 = substantial; 0.81–1.00 = almost perfect) [13].

## Results

### Protocol creation

#### Patient recruitment and language evaluation

**Fig. 1:**
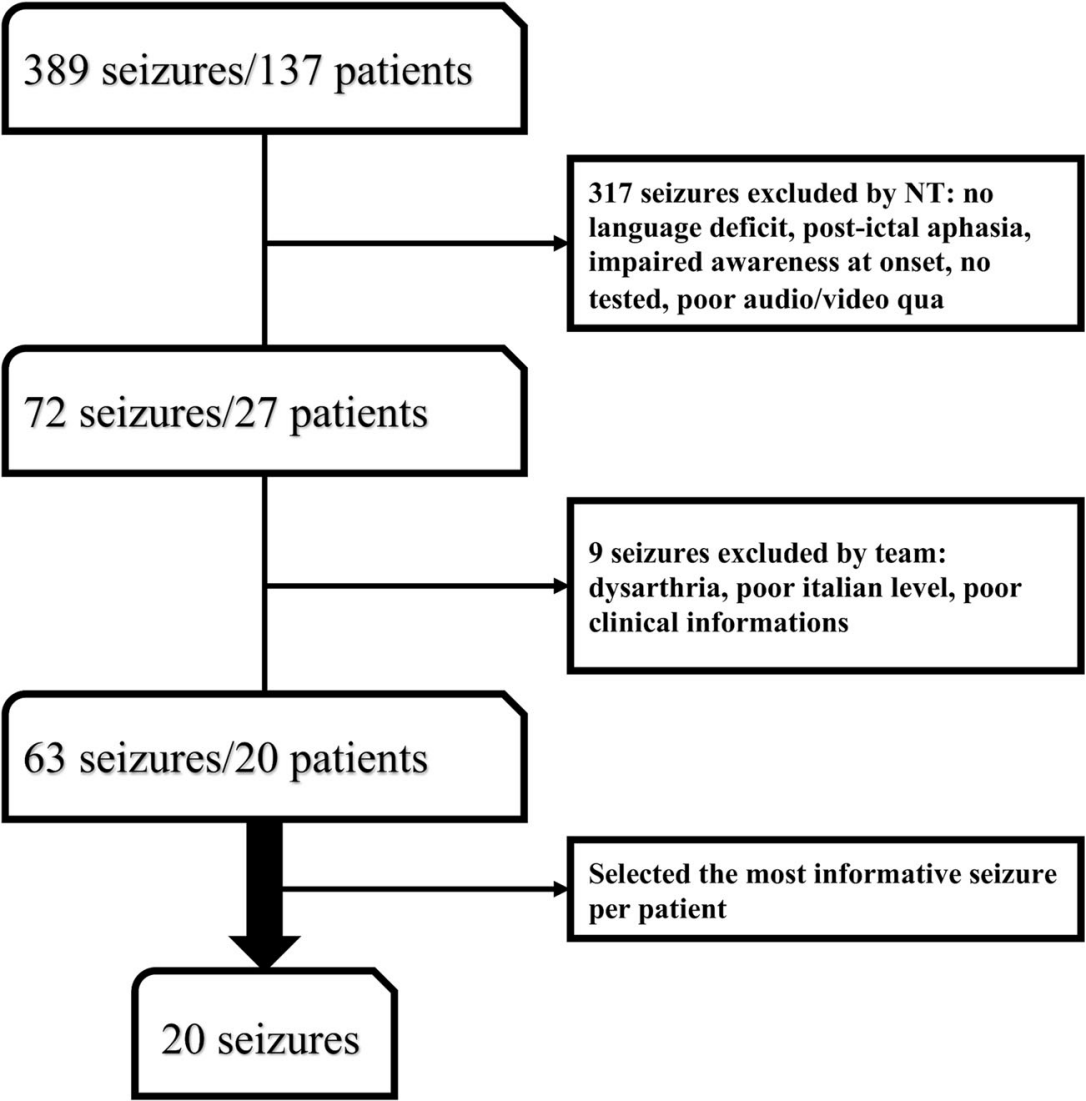
Flowchart of included patients

From our database of 389 ictal VEEG recordings of 137 patients, 72 recordings of 27 patients were selected. According to the inclusion criteria, 63 ictal VEEG recordings from 20 patients (8 males and 12 females) with a mean age of 37.7 years (range: 23–75 years) were considered for the study (Fig. 1). All seizures were tested by EEG technicians. Of the 20 most informative seizures selected by the speech therapists, 12 episodes were classified as A or B, while in the others, the presence, the nature, and the severity of language disturbance could not be characterized (Table 2). According to speech therapist analysis, the main factors limiting an accurate assessment of aphasia were the short duration of the episode, the presence of psychomotor agitation during the seizure, fluctuating contact/awareness, and inappropriate ictal testing.

**Table 2:** Classification of ictal language deficit after communicative interaction analysis

Group A: Defined language deficit (8pt)	
▪ Oral comprehension impairment: 2 patients (4, 5) ▪ Oral production impairment: 8 patients (3,4,5,6,9,12,16,17) a) lexical deficit in 5 pts b) phonological deficit in 1 pt c) lexical and phonological deficit in 1 pt d) poor language sample in 1 pt	
Group B: Probable language deficit (4pt)	
▪ Oral comprehension impairment: 1 patient (15) ▪ Oral production impairment: lexical deficit in 3 patients (7,13,18)	
Group C: No conclusion (8pt)	

#### Protocol development

The multidisciplinary team evaluated the included ictal data and proposed a protocol (Table 3). The protocol starts evaluating oral fluency; subsequently, verbal comprehension and imitation are tested to differentiate comprehension impairment from impaired awareness; lastly, language tests that explore multiple cognitive functions (visual, auditory, attention, praxia) are used to characterize the potential language deficit or/and associate cognitive deficits. This led to the development of a preliminary version of the protocol, incorporating six language tests. To make best use of the time available for testing, the protocol was structured with language testing in hierarchical succession. We decided to incorporate a threat reflex, as this could be a useful test to study visual contact. Besides, we included the recalling of the questions and objects presented during ictal testing in order to test memory retrieval. Lastly, we created the English acronym CA-P-S C-A-R-E to facilitate protocol learning and administration.

**Table 3:** Critical points of examination and correct behavior suggested by logopedic analysis

*• Some patients with fluctuating contact because of attention deficit were tested at onset with denomination or other tests that require multiple cognitive functions* Correct behavior: call the patient by name and place yourself in front of the patient before performing language tests	
*• Simple order given simultaneously by verbal and gestural support* Correct behavior: simple orders must be given by verbal request, if the patient does not respond to two simple orders, try to test comprehension by imitation	
*• Oral production test at onset by means of color denomination or asking to denominate the number of the finger* Correct behavior: to test oral production use closed questions and open questions. The denomination, especially of colors and numbers, should be requested with a simple question, considering that it requires multiple cognitive functions.	
*• Repetition of the same simple question when the patient did not response* Correct behavior: change the simple question, try to simplify it by keeping the semantic value of the question or try to ask a confirmatory question	
*• Repetition of the same question during production or denomination perseveration* Correct behavior: in the presence of verbal perseveration, change the question or the object showed	

#### Protocol description

The protocol is composed of seven simple tests in hierarchical succession, summarized in the acronym CA-P-S C-A-R-E: Closed Answers, Pro-speak question, Simple order, Common object denomination, Audio repetition, Reading, and Evoke. Before proceeding to ictal testing, the examiner must administer the protocol during inter-ictal period to make sure that the patient understands the process and that no language deficits exist during baseline condition.

It was decided to structure the protocol starting from oral production through closed (CA) and open (P) questions, in order to collect an adequate language sample to highlight early paraphasic errors.

Comprehension is tested by means of simple orders (S) given orally and, if compromised, by imitation, in order to rule out a possible awareness impairment. If one of the above tasks is failed, it may be administered a second time in a different manner before moving forward. If comprehension by imitation is impaired, the examiner should continue to give simple motor orders, visually stimulate the patient, and ask closed questions. Comprehension should be re-tested if impaired awareness is suspected during the administration of the following tasks.

**Fig. 2:**
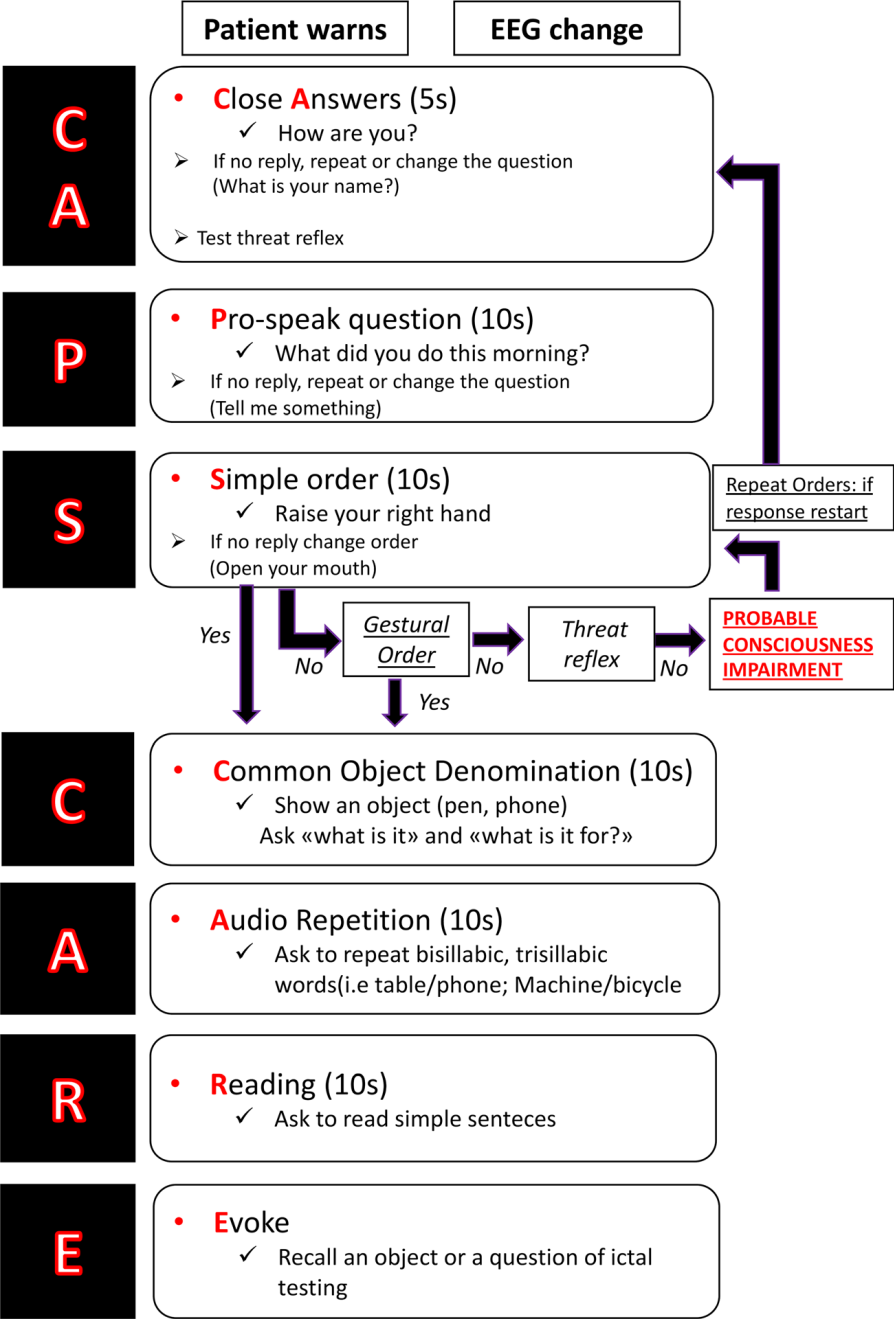
CA-P-S C-A-R-E graphical resume of protocol

Language skills requiring higher cognitive functions are tested by means of denomination of common/daily used object (C), repetition of disyllabic and trisyllabic words (A), and reading of simple sentences (R). When the patient totally recovers from ictal symptoms, namely, he/she is able to perform all the above tasks, the recalling (E) of the questions and objects presented during ictal testing could be used to test memory. In case of ictal speech arrest, the recalling test could also help to differentiate an incoming impaired awareness (i.e., the patient remembers only the first part of the examination), an ictal anarthria (i.e., patient known the answer/object but could not move the month) and ictal aphasia (i.e., patients did not understand the commands or could not find the correct words). A graphical resume of the protocol is represented in Fig. 2. The administration time of the protocol at baseline condition, excluding the recalling test, is less than 1 min, with the first three tests that must be administered in less than 25 s. The full protocol administration instructions and a sample video can be found as supplementary materials.

### Protocol evaluation and interobserver reliability

For assessment of interobserver reliability, we used ten seizures (5M, 5F) tested with the developed protocol. In all cases, the first three steps of the protocol (CA-P-S) were administered within 25 s. The multidisciplinary team reviewed the VEEG recordings and found a language disturbance in three seizures: a production deficit in two cases, followed in one by impaired awareness, and a mixed production and comprehension deficit in the other. In all these cases, the protocol administration was considered accurate in characterizing the language deficit. Six patients had impaired awareness without ictal aphasia, while the other did not show any disturbances.

The video recordings were independently reviewed by 16 different observers: 9 residents in neurology, 2 neurophysiological technicians, 1 biologist, and 4 experienced epileptologists. The overall proportion of agreement in distinguishing between language impairment and other conditions (impairment of awareness, no impairment) was 76%, corresponding to a “substantial” interobserver reliability (kappa 0.61, 95% confidence interval 0.43–0.79).

## Discussion

We developed an original protocol for ictal language evaluation summarized in the acronym CA-P-S C-A-R-E that means Closed Answers, Pro-speak question, Simple orders, Common object denomination, Audio repetition, Reading, Evoke. The protocol was the result of a multi-step process that benefitted from a specific logopedic analysis of VEEG recordings of seizures with ictal aphasia.

Our study confirmed that the recognition of ictal aphasia and its differentiation from impaired awareness is difficult even for expert clinicians, as with 60% of patients studied with logopedic analysis, it was not possible to fully characterize the ictal language deficit. Beyond the intrinsic limitations of ictal assessment, such as the very brief duration or the overlap of multiple symptoms, heterogeneity in test choice, their sequence of administration, and the lack of formation in language evaluation could significantly affect the efficacy of ictal language examination, leading to a loss of useful semiology information and resulting in a substantial inter-operator/unit variability. To address these critical issues, we elaborated a standardized protocol, effective at testing language in a short period of time. This is particularly important in the pre-surgical evaluation for epilepsy surgery, as an early ictal language impairment may suggest an overlap between the language symptomatogenic zone and the epileptogenic zone. The use of the developed protocol in tested patients allowed the distinction between impaired awareness and ictal aphasia in all seizures. In one case, it was even possible to detect an early language deficit before ictal propagation and consequent impaired awareness. The protocol effectiveness likely benefitted from the laboratory VEEG setting, in which there is a close interaction between patient and examiner. This setting facilitated the recognition of aphasic features that may be easily overlooked if the seizures would be tested late. It was decided to test the protocol interobserver reliability for differentiating between ictal aphasia and awareness impairment, as the full language deficit characterization was challenging even for the expert multidisciplinary team. There was substantial agreement found among a heterogeneous population of health professionals with various degrees of experience, suggesting that the employment of our standardized battery might also reduce the inter-observer variability in differentiating ictal aphasia and awareness impairment in examiners without specific training in language evaluation.

A few studies aimed to standardize testing for ictal language examination. Among them, Loesch et al. proposed a battery of five tasks (remember the word, tell me your name, raise both arms, denominate an object, repeat questions 2–4 until full recovery) [14], while Trebuchon and colleagues demonstrated the usefulness of an experience-based protocol for ictal testing, which was well-structured and easy to use [6].

Even if these protocols are quite complete, we believe that our protocol allows a more comprehensive language evaluation. In order to collect an adequate language sample and facilitate recognition of early dysphasic features, open-ended questions should be administered first. Moreover, both previous protocols do not include simple orders by imitation, which are fundamental to discriminate a comprehension deficit from impaired awareness. The latter is a primary cause of reduced quality of life in epileptic patients [15] and is also considered one of the main criteria used to classify focal seizures [16]. For this reason, different authors attempted to develop specific scales and ictal tests to characterize the level and the content of consciousness during the seizure and in the post-ictal period [17–19]. Overall, these approaches improve the characterization of ictal semiology and seizure classification but are still not widely implemented and mostly did not take language into account.

The limitations of our study were the small number of ictal video-EEG recordings included in the analysis, the small number of ictal aphasia recordings tested with the developed protocol, the need of a fast and close interaction between patient and examiner to ensure the evaluation efficacy, and choice of the tests and protocol sequence based on single-center experience.

## Conclusion

We proposed a protocol made by simple and easy to memorize tests to evaluate language and multiple cognitive functions (visual, auditory, praxia, attention, awareness, memory) during a seizure in the shortest possible time, thus helping the examiners to characterize transient language deficits and differentiate them from impaired awareness. We suggest that it should be used to standardize ictal examination especially in epilepsy monitoring units (EMUs) in order to minimize inter-examiner variability. The proposed ictal testing battery should be validated prospectively in different epilepsy centers.

## Supplementary Information

ESM 1 (PDF 130 kb)

ESM 2 (DOCX 19 kb)

ESM 3 (MOV 23191 kb)

ESM 4 (PDF 3585 kb)

## Data Availability

Not applicable.
