# Supplementary material for: If seizures left speechless: CA-P-S C-A-R-E, a proposal of a new ictal language evaluation protocol
Source: Neurol Sci. 2020 Nov 27;42(8):3249–55. doi: 10.1007/s10072-020-04872-x (PMC8342325; doi:10.1007/s10072-020-04872-x)
Supplement: Supplementary file 1 — (PDF 130 kb) [file 10072_2020_4872_MOESM1_ESM.pdf]

CA-P-S C-A-R-E: Full instructions

Patient instructions: before starting video-EEG monitoring the patient should be instructed to warn the technician about seizure start (typical prodromal or other subjective symptoms) possibly by verbal channel, if this is not possible, instruct the patient to push the alarm-button or give some physical signal. Another possible scenario of seizure onset consists in an EEG paroxysmal change without any behavioural or verbal change of the patient. Before performing video-EEG monitoring a baseline examination with the proposed protocol must be obtained; the total optimum time for protocol administration should be shorter than 1 minute. Patient have to be instructed to try to remember the object or question administered during examination.

Oral production evaluation (15 s): if the patient warns verbally about the seizure start, the examiner should encourage oral production by means of one closed question (i.e. "what is your name") and open question (i.e. "What did you do this morning"). If the patient warns by gestures or EEG paroxysmal change is seen on the monitor, the examiner should administer at least two closed questions and an open question. If the patient speaks fluently or a sufficient language sample is available, the examiner must proceed to test comprehension. If the patient does not reply to any questions and more than 15 seconds have elapsed, proceed to test comprehension.

-*Suggestions*: in this first part it is important to draw the attention, placing oneself in front of the patient, calling them by name and testing the visual field (threat reflex) in order to go beyond attention deficit or fluctuating contact.

-*Considerations:* Open and closed questions are administered as first item because it is the simplest and fastest way to point out early dysphasic features.

Oral comprehension evaluation (10 s): test comprehension by means of simple orders (i.e. open the eyes, lift the right arm, open your mouth or push the button). At least two simple orders should be administered. If the patient executes correctly the examiner proceeds with denomination. If the patient does not execute, the examiner has to repeat the same order by imitation; if the patient fulfils the request the examiner proceeds to denomination. If the patient provides some kind of response, after a period of unresponsiveness, the oral production and comprehension should be retested before proceeding to denomination.

-*Suggestions*: in this phase the examiner should passively move the limb of the patient in order to identify hypertonia. When the patient does not fulfil the order even by imitation, change the order, test threat reflex and continue to stimulate the patient with order and simple question.

-*Considerations:* in the event of non-execution of the order by verbal channel and a correct performance by imitation it is possible conclude that a comprehension deficit is present. If the patient does not fulfil the order by imitation an impaired consciousness in probable; to exclude a

condition that could mimic impaired consciousness (i.e. tonic paralysis) the examiner may stimulate other response to the environment (i.e visual track) and retrieval the order during recalling task.

Higher language skills evaluation:

Denomination (10 s): The examiner has to present a pen, a watch or a key and ask to denominate them. If the patient correctly replies the examiner asks what the object is for and if the patient correctly describes the function, the examiner proceeds to test repetition. If the patient denominates mistakenly or cannot describe the function, the examiner presents a second object. If the patient continues to erroneously denominate, the examiner proceeds to repetition. If the patient does not respond, the examiner tries to ask a confirmatory question (i.e "Is this a pen?" showing a watch) in order to retest comprehension. If the patient correctly replies, the examiner proceeds to test repetition, if does not, test comprehension with simple orders and threat reflex to rule out impaired awareness.

-*Suggestions:* Use common objects for denomination tests, possibly daily use objects, perceptively clear and relate to very frequently used words.

-*Considerations*: Denomination allows to better characterize language impairment in terms of phonological or lexical deficit. Besides it allows to testing visual, auditory and long-term memory integrity altogether.

Repetition (10 s): the examiner should use two different words and a short sentence (i.e the dog is hungry) and ask to repeat it. Whether the patient correctly responds or not , the examiner passes to test reading.

-*Suggestion:* use common disyllabic, trisyllabic words and a simple sentence.

-*Considerations*: Repetition allow to identify a conduction aphasia or trans-cortical aphasia.

Reading (10 s): the examiner should invite the patient to read simple sentences (i.e. short sentences of NIH Stroke Scale) or few lines from a book/newspaper.

-*Suggestions:* reading could be performed also in the post-ictal phase, until the full recovery of language.

-*Considerations:* reading is used to better characterize semantic or phonemic paraphasia, identify specific sub-aphasic form (alexia) or visual agnosia.

Evoke: When the patient totally recovers from ictal symptoms, namely, he/she is able to perform all the above tasks, the examiner should ask for recall a question and an object presented during ictal testing.

-*Suggestions:* It would be advisable to recall either an item altered during seizure than a normal one.

-*Considerations:* recalling it use to investigate memory deficits; it could also help to discriminate incoming impaired awareness (i.e. the patient remember just the first item but not the second).
