# Supplementary material for: If seizures left speechless: CA-P-S C-A-R-E, a proposal of a new ictal language evaluation protocol
Source: Neurol Sci. 2020 Nov 27;42(8):3249–55. doi: 10.1007/s10072-020-04872-x (PMC8342325; doi:10.1007/s10072-020-04872-x)
Supplement: Supplementary file 2 — (DOCX 19 kb) [file 10072_2020_4872_MOESM2_ESM.docx]

| **Pt** | **Sex** | **Mother tongue** | **Handedness** | **School.** | **Age at onset** | **Age at obser.** | **MRI** | **Inter-ictal EEG** | **N. of seizure rec.** | **Ictal EEG** | **NPS test** |
| --- | --- | --- | --- | --- | --- | --- | --- | --- | --- | --- | --- |
| **1** | F | IT | rh | 16 | 14 | 26 | Left fronto-mesial and pre-rolandic dysplasia | Bilateral fronto-temporal | 16 | Diffuse discharge prominent on left fronto-central | n.a. |
| **2** | F | P | rh | 13 | 2.5 | 39 | Post-traumatic rh temporo-basal malacic sequelae | Bilateral fronto-temporal anomalies syncrounous/asyncronous rh>lf | 2 | Recruitment activity rh frontal | n.a. |
| **3** | M | IT | rh | 13 | 20 | 25 | normal | Bilateral asyncronous temporal anomalies lf>rh | 2 | Rh fronto-temporal discharge with contralateral diffusion | IQ at lower normal limit |
| **4** | M | IT | rh | 15 | 11 | 28 | Left tempro-mesial dysplasia | Left temporal | 7 | Left temporal discharge | Normal |
| **5** | F | IT | rh | 8 | 65 | 75 | Chronic ischemic encephalopathy | Left fronto-temporal | 3 | Left fronto-temporal discharge | n.a. |
| **6** | F | IT  | rh | 13   | 16  | 43  | Left parietal atrophy | Left temporo-parietal occipital anomalies | 8 | Left temporo-parietal occipital discharge | normal |
| **7** | F | IT | rh | 8 | 38 | 42 | Right temporo-frontal dysplasia | Bilateral asynccronous anomalies fronto-temporal rh>lf | 2 | Posterior right temporal discharge | normal |
| **8** | F  | IT   | lf | 13 | 20 | 44 | Suspect Right temporo-mesial dysplasia | Bilateral temporal anomalies lf>rh | 2 | Left temporal discharge | Memory consolidation deficit |
| **9** | F | IT | rh | 13 | 22 | 24 | Left temporal cavernoma | Left temporal anomalies | 4 | Left temporal discharge | Phonemic fluency and verbal logic deficit |
| **10** | M | IT | rh | 18 | 17 | 25 | Right amygdala dysplasia | Aspecific right temporal anomalies | 3 | Diffuse right hemispheric discharge with contralateral diffusion | Normal |
| **11** | F | IT | rh | 13 | 29 | 58 | Right posterro-insular dysplasia | Aspecific anomalies right centro-parietal | 2 | Flattening background activity rh>lf | n.a |
| **12** | M | IT | rh | 18 | 45 | 58 | Normal | Bilateral temporal anomalies lf>rh | 1 | Left temporal discharge | n.a |
| **13** | F | IT | rh | 13 | 16 | 36 | Normal | Bilateral temporal anomalies rh>lf | 1 | Left temporal discharge with contralateral diffusion | n.a |
| **14** | F | IT | rh | 8 | 9 | 33 | Left mesial temporal sclerosis | Bilateral temporal anomalies lf>rh | 1 | Left temporal discharge | IQ at lower normal limit |
| **15** | F | IT | rh | 13 | 1,2 | 23 | Left temporal dysplasia | Left temporal anomalies | 1 | Left temporal discharge with contralateral diffusion | n.a |
| **16** | M | IT | rh | 13 | 16 | 30 | Right temporo-parietal-occipital gliosis | Normal | 1 | Right fronto-temporal-parietal discharge | normal |
| **17** | M | IT | rh | 13 | 34 | 36 | Left temporo-polar cavernous hemangioma | Left temporal anomalies | 1 | Left temporal discharge | normal |
| **18** | M | IT | Lf | 16 | 17 | 39 | Normal | Diffuse anomalies left occipital and right temporal | 1 | Left posterior discharge | n.a |
| **19** | F | IT | rh | 13 | 20 | 34 | Rightmesial temporal sclerosis | Bilateral temporal anomalies rh>lf | 3 | Right temporal discharge | normal |
| **20** | M | IT | rh | 18 | 25 | 35 | Right sphenoidal bone-dysplasia | Left temporal anomalies | 2 | Left temporal discharge | normal |

**Table 1:** Main anatomo-electro-clinical data of patient included in logopaedic analysis.
